# Restoring Control: Real-World Success with Imipenem–Relebactam in Critical MDR Infections—A Multicenter Observational Study

**DOI:** 10.3390/pathogens14070685

**Published:** 2025-07-11

**Authors:** Andrea Marino, Giuseppe Pipitone, Emmanuele Venanzi Rullo, Federica Cosentino, Rita Ippolito, Roberta Costa, Sara Bagarello, Ylenia Russotto, Chiara Iaria, Bruno Cacopardo, Giuseppe Nunnari

**Affiliations:** 1 Department of Clinical and Experimental Medicine, Unit of Infectious Diseases, ARNAS Garibaldi Hospital, University of Catania, 95122 Catania, Italy; andrea.marino@unict.it (A.M.); federicacosentino91@gmail.com (F.C.); costa.roberta@outlook.it (R.C.); cacopardobruno@inwind.it (B.C.); giuseppe.nunnari1@unict.it (G.N.); 2 Infectious Diseases Unit, ARNAS Civico-Di Cristina, 90127 Palermo, Italy; sbagarello@gmail.com (S.B.); iaria.chiara@gmail.com (C.I.); 3 Unit of Infectious Diseases, G. Martino University Hospital, Department of Clinical and Experimental Medicine, University of Messina, 98124 Messina, Italy; emmanuele.venanzirullo@unime.it (E.V.R.); rita.ippolito29@gmail.com (R.I.); ylenia.russ@gmail.com (Y.R.); 4 Infectious Diseases Unit, University Hospital P. Giaccone, 90127 Palermo, Italy

**Keywords:** imipenem-relebactam, Enterobacterales, MDR bacteria, antimicrobial stewardship, *Klebsiella pneumoniae*, *Pseudomonas aeruginosa*

## Abstract

**Background**: Multidrug-resistant (MDR) Gram-negative infections, particularly those caused by carbapenem-resistant *Enterobacterales* (CRE) and difficult-to-treat *Pseudomonas aeruginosa* (DTR-Pa), present a growing global healthcare challenge, especially in critically ill populations. Imipenem–relebactam (I/R), a novel β-lactam/β-lactamase inhibitor combination, has shown efficacy in clinical trials, but real-world data remain limited. **Methods**: We conducted a multicenter, retrospective–prospective observational study across tertiary-care hospitals in Italy between January 2020 and May 2025. Adult patients (≥18 years) treated with I/R for ≥48 h for suspected or confirmed MDR Gram-negative infections were included. Primary endpoints were clinical success at the end of therapy and 30-day all-cause mortality. Secondary endpoints included microbiological eradication, recurrence, safety, and predictors of treatment failure. Statistical analysis involved descriptive methods and correlation analysis for mortality predictors. **Results**: Twenty-nine patients were included (median age 66 years; 58.6% ICU admission; 71.4% mechanical ventilation). Clinical success was achieved in 22/29 patients (75.9%), while 30-day mortality was 24.1% (7/29). The most common pathogen was *Klebsiella pneumoniae* (62.1%), with 41.4% of infections being polymicrobial. Microbiological eradication was confirmed in all the BSIs. Parenteral nutrition (*p* = 0.016), sepsis at presentation (*p* = 0.04), candidemia (*p* = 0.036), and arterial catheter use (*p* = 0.029) were significantly more frequent in non-survivors. Survivors showed significant reductions in CRP, PCT, and bilirubin at 48 h, while non-survivors did not. Parenteral nutrition (rho = 0.427, *p* = 0.023), sepsis (rho = 0.378, *p* = 0.043), and arterial catheter use (rho = 0.384, *p* = 0.04) were significantly correlated with mortality. **Conclusions**: In this Italian multicenter cohort of critically ill patients, imipenem–relebactam demonstrated high clinical success and acceptable mortality rates in the treatment of severe MDR Gram-negative infections, particularly those caused by KPC-producing *K. pneumoniae*. Early biomarker dynamics may aid in monitoring treatment response. Larger prospective studies are needed to confirm these findings and define optimal treatment strategies.

## 1. Introduction

The escalating global crisis of multidrug-resistant (MDR) Gram-negative infections poses a formidable challenge to modern healthcare, particularly within critical care environments where patient vulnerability is heightened, leading to increased risks of complications and adverse outcomes [1]. Among the most critical threats are carbapenem-resistant Enterobacterales (CRE) and difficult-to-treat *Pseudomonas aeruginosa* (DTR-*Pa*) infections, which are associated with severely limited therapeutic options and alarmingly high mortality rates [2,3]. The development of novel antimicrobial agents has been crucial in addressing these challenges [4,5]. Imipenem–relebactam, a combination of a well-established carbapenem and a novel diazabicyclooctane β-lactamase inhibitor, offers a promising therapeutic avenue by effectively targeting resistance mechanisms mediated by Ambler class A (such as KPC), class C (AmpC), and certain class D β-lactamases (like OXA-48-like) [6,7].

The efficacy and safety of imipenem–relebactam have been established in pivotal clinical trials. The RESTORE-IMI 1 trial [8] demonstrated its superiority over colistin plus imipenem for treating imipenem-resistant bacterial infections, while the RESTORE-IMI 2 trial [9] confirmed its non-inferiority to piperacillin–tazobactam for hospital-acquired or ventilator-associated pneumonia (HAP/VAP). These trials provided essential evidence for regulatory approval and initial clinical guidance.

However, the transition from controlled clinical trial settings to real-world clinical practice often reveals a broader spectrum of patient complexities and treatment scenarios [10]. Real-world data on imipenem–relebactam, especially from diverse geographical regions and patient populations—including those with polymicrobial infections, profound comorbidities, extended Intensive Care Unit (ICU) admissions, or prior treatment failures—remains somewhat limited, though continuously emerging [10,11]. Observational studies are therefore indispensable for comprehensively understanding the effectiveness and safety of imipenem–relebactam across these varied and often more challenging clinical situations.

This study aimed to evaluate the real-world effectiveness and safety of imipenem–relebactam in a multicenter cohort of patients presenting with suspected or confirmed MDR Gram-negative infections. The primary focus was on assessing clinical and microbiological outcomes, 30-day mortality, and identifying potential predictors of treatment failure in a complex patient population.

## 2. Materials and Methods

### 2.1. Study Design and Setting

This was a multicenter, retrospective–prospective observational cohort study conducted in three tertiary-care hospitals across Sicily (Italy): Catania (ARNAS Garibaldi Hospital), Messina (Policlinico “G. Martino”), and Palermo (ARNAS Ospedali Civico Di Cristina). The study period extended from January 2020 to May 2025. This investigation was designed to evaluate the real-life effectiveness and safety of imipenem–relebactam in adult patients with documented or suspected infections due to multidrug-resistant (MDR) Gram-negative bacteria. Data were collected through electronic medical records and clinical chart reviews.

### 2.2. Inclusion and Exclusion Criteria

Patients were eligible for inclusion if they met all of the following criteria:

- Age ≥ 18 years.
- Treated with imipenem–relebactam for ≥48 h.
- Documented or suspected infection caused by Gram-negative MDR pathogens, including the following:
  ○ Complicated intra-abdominal infections (cIAIs);
  ○ Complicated urinary tract infections (cUTIs);
  ○ Hospital-acquired pneumonia (HAP) or ventilator-associated pneumonia (VAP);
  ○ Bloodstream infections (BSIs) due to pathogens susceptible to imipenem–relebactam.
- Availability of complete microbiological and clinical outcome data.
- Patients were excluded based on the following criteria:
- Received imipenem–relebactam for <48 h.
- Had incomplete clinical documentation.
- Had co-infections with pathogens resistant to imipenem–relebactam that were not covered by concurrent antimicrobial therapy.
- Were pregnant, breastfeeding, or receiving palliative care.
- Had a life expectancy <48 h or <30 days due to non-infectious causes.

*Data Collection*

Demographic, clinical, and microbiological data were collected using a standardized case report form. Variables recorded included the following:

- Demographics (age, sex);
- Comorbidities (e.g., diabetes, chronic kidney disease, cardiovascular disease);
- Charlson Comorbidity Index (CCI);
- Infection type and source;
- ICU admission and duration;
- Use of invasive devices (mechanical ventilation, arterial catheters);
- Antimicrobial regimen details (dose, duration, concurrent therapies);
- Laboratory parameters (CRP, PCT, bilirubin, platelets, renal function) at baseline and after 48 h of therapy.

### 2.3. Definitions

For the purpose of this study, multidrug-resistant (MDR), extensively drug-resistant (XDR), and pandrug-resistant (PDR) pathogens were defined according to the international expert proposal for interim standard definitions for acquired resistance [12].

A “confirmed infection” was defined as the administration of imipenem–relebactam following the isolation of a Gram-negative pathogen with a susceptibility profile indicating its appropriateness (targeted therapy). A “suspected infection” was defined as the empirical initiation of imipenem–relebactam for a severe infection (e.g., sepsis or septic shock) in patients with significant risk factors for harboring an MDR Gram-negative pathogen, such as known prior colonization with carbapenem-resistant Enterobacterales (CRE), recent hospitalization, or prior antimicrobial failure, pending final microbiological results [13]. Sepsis and septic shock were defined according to the Third International Consensus Definitions for Sepsis and Septic Shock (Sepsis-3) criteria [14].

### 2.4. Outcomes

Primary endpoints:

1. Clinical success at the end of therapy, defined as complete resolution or significant improvement in signs and symptoms of infection without the need for modification of antibiotic therapy.
2. Thirty-day all-cause mortality, defined as death from any cause within 30 days from initiation of imipenem–relebactam.

Secondary endpoints:

- Microbiological eradication, defined as documented negative cultures following completion of therapy.
- Duration of hospital and ICU stay.
- Infection recurrence or re-infection within 30 days of treatment completion.
- Incidence of therapy-related adverse events.
- Associations between demographical/clinical factors and mortality.

### 2.5. Microbiological Evaluation

All microbiological testing was performed according to local laboratory protocols. Pathogen identification was performed using standard laboratory methods such as MALDI-TOF MS. Antimicrobial susceptibility testing (AST) was conducted using automated systems (e.g., VITEK^®^ 2 (Biomerieux-Marcy-l’Étoile, France), Phoenix™ (Becton, Dickinson and Company-Franklin Lakes, NJ, USA)) or broth microdilution, with results interpreted in accordance with the European Committee on Antimicrobial Susceptibility Testing (EUCAST) breakpoints for the relevant year [15]. Molecular testing for carbapenemase production (e.g., KPC, OXA-48, NDM) was performed at the discretion of the individual centers, reflecting real-world variations in diagnostic workflows; these data were available for a subset of isolates from patients with carbapenem-resistant Enterobacterales.

### 2.6. Statistical Analysis

Descriptive statistics were used to summarize patient characteristics and outcomes. Continuous variables were reported as medians with interquartile ranges (IQRs) and compared using the Mann–Whitney U test. Categorical variables were expressed as counts and percentages, with comparisons made using Fisher’s exact or Chi-square test as appropriate. Spearman’s rho correlation was performed to identify risk factors associated with 30-day mortality. All analyses were performed using SPSS (c) (v 21).

### 2.7. Ethical Considerations

This study was conducted in accordance with the principles of the Declaration of Helsinki and Good Clinical Practice guidelines. The study protocol was reviewed and approved by the local Ethics Committee Comitato Etico Catania 2 (approval n°101/CECT2).

Given the retrospective and observational nature of this study, the need for written informed consent was waived by the Ethics Committees, provided that all data were anonymized and handled in compliance with national privacy laws (Legislative Decree 196/2003 and EU Regulation 2016/679-GDPR). For prospective data collection, informed consent was obtained from all patients or their legal representatives whenever required by local regulations.

## 3. Results

### Study Population

A total of 29 patients treated with imipenem–relebactam between January 2020 and May 2025 were included. The median age was 66 years (IQR 57–74.5), and 55.2% were male. The most frequent comorbidities were chronic kidney disease (31%), coronary heart disease (37.9%), and diabetes mellitus (27.6%). The median Charlson Comorbidity Index was 4 (IQR 2–6). Patients received a median of 11 days of therapy (IQR 7.5–14.4) and had a median hospital stay of 48 days (IQR 29.5–89.5). Targeted therapy was administered in 21/29 (72.4%) of patients.

Seventeen patients (58.6%) required ICU admission, and twenty of twenty-eight evaluated (71.4%) underwent mechanical ventilation (MV). The median duration of MV was 26 days (IQR 5–37) overall but was significantly longer among those who died (34.5 vs. 12 days, *p* = 0.013) (Table 1).

*Primary Endpoints*

- **Clinical Success**: A total of 22 of 29 patients (75.9%) achieved clinical success at the end of the therapy. Early clinical improvement within 72 h was observed in 14 of 28 evaluable patients (50%).
- **Thirty-day Mortality**: Seven patients (24.1%) died within 30 days of therapy initiation. Compared to survivors, non-survivors had significantly longer MV duration (34.5 vs. 12 days, *p* = 0.013) and higher rates of sepsis at presentation (100% vs. 54.5%, *p* = 0.04), candidemia (71.4% vs. 27.3%, *p* = 0.036), parenteral nutrition (83.3% vs. 28.6%, *p* = 0.016), and arterial catheter use (85.7% vs. 38.1%, *p* = 0.029) (Table 1).

*Secondary Endpoints*

- **Microbiological Eradication**: Among 27 patients with positive cultures, 10/27 (30%) had positive blood cultures. Among those patients with BSI, the median time to culture negativization was 10 days (IQR 5.25–12.25) with no significant difference between survivors and non-survivors (*p* = 0.46). Overall eradication was confirmed in all patients with BSI.
- **Infections and Pathogens***:* Hospital-acquired infections predominated (82.8% vs. 13.8% community-acquired). *Klebsiella pneumoniae* was the most common isolate (18/29, 62.1%), followed by *Pseudomonas aeruginosa* (3/29, 10.3%). Polymicrobial infections occurred in 12 of 27 patients (44.4%), and 16 of 28 (57.1%) were colonized with KPC-producing microorganisms prior to infection.
- **Recurrence and Re-infection***:* Three out of twenty-three patients (13%) experienced recurrence within 30 days of treatment completion, with a higher percentage among non-survived patients compared to alive patients (25% vs. 10.5%, respectively) without statistically significant differences (Table 2).

**Table 2:** Primary and secondary endpoints of the overall cohort. ATB: antibiotics; RS: rectal swab.

	Overall (n = 29)	Dead (n = 7)	Alive (n = 22)	*p*-Value
CA infection	4/29 (13.8%)	2/7 (28.6%)	2/22 (9.1%)	0.193
HA infection	24/29 (82.8%)	6/7 (85.7%)	18/22 (81.8%)	0.8
MV	20/28 (71.4%)	6/6 (100%)	14/22 (63.6%)	0.08
ECMO	1/28 (3.6%)	1/6 (16.7%)	0/22	**0.051**
Parenteral nutrition	11/27 (40.7%)	5/6 (83.3%)	6/21 (28.6%)	**0.016**
Dialysis	5/28 (17.9%)	1/6 (16.7%)	4/22 (18.2%)	0.93
Central vein	25/28 (89.3%)	7/7 (100%)	18/21 (85.7%)	0.29
CVC	22/28 (78.6%)	7/7 (100%)	15/21 (71.4%)	0.11
Arterial catheter	14/28 (50%)	6/7 (85.7%)	8/21 (38.1%)	**0.029**
PICC/midline	15/28 (35.6%)	2/7 (28.6%)	13/21 (61.9%)	0.13
PORT-a-Cath	2/29 (6.9%)	0/7	2/22 (9.1%)	0.4
KPC colonized (RS)	16/28 (57.1%)	6/7 (85.7%)	10/21 (47.6%)	0.078
COVID-19	1/29 (3.5%)	0/7	1/22 (4.5%)	0.5
Sepsis	19/29 (65.5%)	7/7 (100%)	12/22 (54.5%)	**0.04**
Shock	16/29 (55.2%)	5/7 (71.4%)	11/22 (50%)	0.32
Polymicrobial isolates	12/27 (44.4%)	4/6 (66.7%)	8/21 (38.1%)	0.33
Isolates type distribution				**0.035**
*K. pneumoniae*	18	1	17	
*P. aeruginosa*	3	1	2	
*S. marcescens*	1	1	0	
*M. morganii*	1	1	0	
Previous ATB	26/26 (100%)	7/7	19/19	0.3
Targeted therapy	21/29 (72.4%)	5/7 (71.4%)	16/22 (72.7%)	0.95
Combination ATB	14/29 (48.3%)	4/7 (57.1%)	10/22 (45.5%)	0.36
Early clinical improvement	14/29 (50%)	2/7 (28.6%)	12/22 (54.5%)	0.23
C. difficile infection	1/29 (3.5%)	0/7	1/22 (4.5%)	0.57
Candidemia	11/29 (37.9%)	5/7 (71.4%)	6/22 (27.3%)	**0.036**
Re-infection	3/23 (13%)	1/4 (25%)	2/19 (10.5%)	0.45

*Laboratory Parameters and Dynamics*

- **Overall Change (T0→T1 at 48 h):** In the entire cohort, median CRP declined from 16.8 to 9.15 mg/dL (*p* = 0.027), and PCT from 3.0 to 1.0 ng/mL (*p* = 0.004). Other parameters, including hemoglobin, WBC, creatinine, bilirubin, and fibrinogen, showed no significant shifts.
- **Survivors:** Among survivors (n = 22), CRP fell from 10.8 to 5.3 mg/dL (*p* = 0.03), PCT from 3.1 to 0.93 ng/mL (*p* = 0.01), and total bilirubin from 1.0 to 0.85 mg/dL (*p* = 0.02). Other markers, including platelet count, did not change significantly.
- **Non-survivors:** No laboratory parameter demonstrated a statistically significant change at 48 h in non-survivors (Table 3).

**Table 3:** Laboratory dynamics (baseline T0→48 h T1). CRP: C-reactive protein.

Parameter	Overall T0 (n = 29)	Overall T1 (n = 29)	*p*-Value	Survivors T0 (n = 22)	Survivors T1 (n = 22)	*p*-Value	Non-Survivors T0 (n = 7)	Non-Survivors T1 (n = 7)	*p*-Value
CRP (mg/dL)	16.8 (6.4–22.7)	9.15 (3.81–20.3)	0.027	10.8 (5.6–20.6)	5.3 (3.2–16.9)	0.03	20.6 (12.2–25.0)	14.9 (10.1–23.0)	0.16
Procalcitonin (ng/mL)	3.0 (0.67–8.67)	1.0 (0.4–5.6)	0.004	3.1 (0.39–11.46)	0.93 (0.34–5.42)	0.01	3.0 (1.94–4.7)	2.26 (0.73–8.9)	0.73
Total bilirubin (mg/dL)	1.0 (0.64–1.48)	0.85 (0.48–1.2)	0.12	1.0 (0.6–1.32)	0.85 (0.43–1.04)	0.02	0.7 (0.67–2.32)	1.1 (0.5–8.7)	1.00
Platelet count (/µL)	180,000 (98,500–317,500)	235,000 (100,500–336,250)	0.93	190,000 (99,250–346,500)	273,500 (135,500–341,500)	0.45	122,000 (82,000–254,000)	95,500 (64,250–155,000)	0.06

Combination therapy, administered to 14/29 (48.3%) patients, was most often used to cover for other pathogens in confirmed polymicrobial infections (44.4% of cases) or as part of an empirical regimen for sepsis in line with institutional protocols, rather than as a specific strategy to enhance I/R activity against the target Gram-negative pathogen (e.g., addition of fosfomycin for DTR-*P. aeruginosa* [16,17]).

The four patients with community-acquired infections had significant healthcare-associated risk factors (e.g., recent hospitalization, residence in a long-term care facility) that prompted broad empirical coverage for MDR pathogens.

*Risk Factors for 30-Day Mortality*

Spearman’s correlation showed a mild positive correlation for parenteral nutrition, arterial catheter, and sepsis with 30-day mortality (Table 4).

## 4. Discussion

The emergence and spread of multidrug-resistant (MDR) Gram-negative microorganisms, particularly carbapenem-resistant Enterobacterales (CRE) like KPC-producing *Klebsiella pneumoniae* and difficult-to-treat *Pseudomonas aeruginosa* (DTR-*P. aeruginosa*), represent a significant global health crisis, especially in critically ill patient populations [1]. Our study contributes to the growing body of real-world evidence on the use of imipenem–relebactam (I/R) in such challenging scenarios. We observed an overall clinical success rate of 75.9% and a 30-day all-cause mortality rate of 24.1% in a cohort of 29 critically ill adult patients treated with I/R for MDR Gram-negative infections, predominantly KPC-*K. pneumoniae* (62.1%). These patients were characterized by high acuity, with a median Charlson Comorbidity Index of 4, 58.6% requiring ICU admission, and 71.4% receiving mechanical ventilation.

We selected I/R because our region has a high prevalence of KPC-producing Klebsiella pneumoniae (~65% of *K. pneumoniae* bloodstream isolates in 2023) [18]. Unlike meropenem–vaborbactam, I/R retains potent activity against *P. aeruginosa* with difficult-to-treat resistance patterns and remains stable in the presence of AmpC and ESBL enzymes; furthermore, the increase in KPC variant rates in our region [19] made the choice of ceftazidime–avibactam challenging. Additionally, the imipenem backbone provides intrinsic activity against *Enterococcus faecalis*, an advantage in our critically ill cohort where mixed Gram-negative/positive infections are common. Meropenem–vaborbactam and ceftazidime–avibactam were available but were reserved when local susceptibility, formulary restrictions, or specific patient factors made them preferable (e.g., ceftazidime–avibactam for infections with ceftazidime-susceptible *P. aeruginosa*). These microbiological, pharmacological, and stewardship considerations collectively justified prioritizing I/R in the present study.

Our findings are situated within a spectrum of outcomes reported in the recent real-world literature, though direct comparisons are nuanced by inherent heterogeneity in study populations, local epidemiology, infection types, and pathogen distribution. For instance, the clinical success rate in our cohort is encouraging when compared to some studies focusing on highly resistant *P. aeruginosa*. The IMRECOR study in Spain, focusing on DTR-*P. aeruginosa* in 14 critically ill patients, reported a clinical efficacy of 64.3% and a 30-day mortality of 42.9% [6]. Similarly, the preliminary results from the US-based MIRAGE study on 32 patients with MDR-*P. aeruginosa* pneumonia and BSIs indicated a 30-day clinical success of 53% and 30-day mortality of 22% in a cohort with high illness severity (81% mechanically ventilated, median SOFA 9) [20]. Another small US study by Vu et al. [21] involving 10 patients with XDR/DTR-*P. aeruginosa* infections (primarily VAP) showed 70% clinical success and 30% 30-day mortality. In a larger US multicenter real-world study by Caniff et al. [11] involving 151 patients (72.2% *P. aeruginosa*), a clinical success rate of 70.2% was reported. While our study had a smaller proportion of *P. aeruginosa* infections (10.3%), the outcomes for this pathogen subset are important to consider in the broader context of I/R utility. These findings align with the efficacy demonstrated in the pivotal RESTORE-IMI 1 [8] and 2 [22] trials, where I/R was found to be effective for various infections, including those caused by imipenem-resistant pathogens and HAP/VAP, and a meta-analysis of RCTs confirmed I/R′s non-inferior efficacy and comparable safety to standard of care [23].

A key strength of our study is its focus on KPC-*K. pneumoniae*, the predominant pathogen in our cohort. Real-world data on I/R for KPC-*K. pneumoniae* infections are still accumulating. A small, single-center Italian study by Leanza et al. [24] reported 100% clinical cure and 0% mortality in 10 patients, including 6 with KPC-*K. pneumoniae* infections, highlighting potentially excellent outcomes in specific settings, although limited by its sample size. Our observed 75.9% success rate provides further support for I/R in this context, especially considering the high baseline severity of our patients. The strong in vitro activity of I/R against KPC-producing *K. pneumoniae*, with susceptibility rates often exceeding 90–97% as reported by several authors [25,26], underpins its clinical utility for these challenging isolates.

The identification of risk factors for mortality is crucial for patient stratification and management. Our Spearman’s correlation analysis, detailed in Table 4, investigated associations with 30-day mortality. This analysis showed that parenteral nutrition (rho = 0.427, *p* = 0.023), arterial catheter use (rho = 0.384, *p* = 0.04), and sepsis at presentation (rho = 0.378, *p* = 0.043) were significantly correlated with increased 30-day mortality. Candidemia demonstrated a positive correlation that approached statistical significance in this analysis (rho = 0.354, *p* = 0.055), whereas mechanical ventilation, when assessed as a variable in the correlation analysis in Table 4, did not show a statistically significant correlation with mortality (rho = 0.315, *p* = 0.096). It is noteworthy, however, that other analyses comparing non-survivors to survivors revealed significantly longer mechanical ventilation duration in non-survivors (*p* = 0.013, as shown in Table 1). Furthermore, higher rates of sepsis at presentation (100% vs. 54.5%, *p* = 0.04, Table 2), candidemia (71.4% vs. 27.3%, *p* = 0.036, Table 2), parenteral nutrition (83.3% vs. 28.6%, *p* = 0.016, Table 2), and arterial catheter use (85.7% vs. 38.1%, *p* = 0.029, Table 2) were also observed in non-survivors. Collectively, these factors—parenteral nutrition, arterial catheter use, sepsis, candidemia, and prolonged mechanical ventilation—often reflect underlying illness severity, prolonged hospitalization, and susceptibility to complications. These findings resonate with predictors identified in larger trials and observational studies. For example, the sub-analysis of the RESTORE-IMI 2 trial [22] identified vasopressor use, renal impairment, bacteremia, and APACHE II scores ≥ 15 as predictors of 28-day mortality in HAP/VAP patients. Caniff et al. [11] reported heart failure, prior antibiotic use, ICU admission at index culture, and DTR-P. aeruginosa as risk factors for reduced clinical success. The inability of our study to identify independent predictors in a multivariate model is likely due to the limited sample size and number of events, a common challenge in studies of novel antibiotics for severe infections. Furthermore, our observation that survivors exhibited a significant decrease in CRP and PCT levels at 48 h post-I/R initiation, unlike non-survivors, suggests the potential utility of these biomarkers in early monitoring of therapeutic response. This dynamic change in inflammatory markers warrants further investigation in larger, prospective studies to establish its predictive value.

A significant portion of our cohort (57.1%) was known to be colonized with KPC-producing bacteria prior to initiating therapy. We strongly concur with the principle that colonization is not an indication for antimicrobial therapy [27,28]. In our study, the decision to initiate imipenem–relebactam was made only when patients developed compelling clinical evidence of a new or worsening infection, most notably sepsis (present in 65.5% of the cohort) or septic shock. Known colonization status was therefore not a trigger for treatment but rather a critical factor in risk-stratifying the patient to guide the selection of an appropriate empirical agent once the clinical need for an antibiotic was established.

The clinical implications of our study are several. It supports the role of I/R as an important therapeutic option for severe MDR Gram-negative infections, including those caused by KPC-*K. pneumoniae* and other carbapenem-resistant bacteria, particularly in critically ill patients with limited alternative treatments. The observed efficacy, combined with the known susceptibility patterns, positions I/R as a valuable agent in antimicrobial stewardship programs aimed at preserving carbapenem efficacy while providing effective therapy.

Nevertheless, our study has several limitations. Its observational design is susceptible to inherent biases, including selection bias. The sample size of 29 patients, while providing valuable insights, is modest and limits the statistical power for more granular analyses, particularly for identifying independent risk factors for mortality. Although multicenter, this study was conducted exclusively in Italy, which may limit the generalizability of our findings to other geographical regions with different healthcare practices or CRE epidemiology. The retrospective data collection for some variables might also introduce bias.

Despite these limitations, our study provides important real-world evidence on the use of I/R in a critically ill patient population in Italy, a country with a high prevalence of KPC-producing CRE. It highlights favorable clinical outcomes and contributes to understanding the drug′s performance outside the controlled environment of clinical trials. Future research should focus on larger, prospective multicenter observational studies to further delineate the effectiveness of I/R across diverse patient populations and geographical regions. Comparative effectiveness studies evaluating I/R against other novel β-lactam/β-lactamase inhibitor combinations would be invaluable. Additionally, research into optimal dosing strategies, duration of therapy, and the potential role of I/R in combination regimens for highly resistant pathogens or in specific clinical scenarios like septic shock is warranted.

## Figures and Tables

**Table 1:** Baseline characteristics of the overall cohort. BC: blood culture; MV: mechanical ventilation; CKD: chronic kidney disease; CHD: coronary heart disease; CCI: Charlson Comorbidity Index.

	Overall (n = 29)	Dead (n = 7)	Alive (n = 22)	*p*-Value
Age (years, IQR)	66 (57–74.5)	59 (55–72)	66.5 (58.25–72.25)	0.72
Sex (M)	16/29 (55.2%)	3/7 (42.9%)	13/22 (59.1%)	0.67
BMI > 30	7/29 (24.1%)	1/7 (14.3%)	6/22 (27.3%)	0.65
COPD	5/29 (17.2%)	1/7 (14.3%)	4/22 (18.2%)	1
CKD	9/29 (31%)	2/7 (28.6%)	7/22 (31.8%)	1
Neoplasm	5/29 (17.2%)	1/7 (14.3%)	4/22 (18.2%)	1
Diabetes	8/29 (27.6%)	3/7 (42.9%)	5/22 (22.7%)	0.36
CHD	11/29 (37.9%)	4/7 (57.1%)	7/22 (31.8%)	0.38
Cirrhosis	2/29 (6.9%)	0/7	2/22 (9.1%)	1
CAD	2/29 (6.9%)	1/7 (14.3%)	1/22 (4.5%)	0.43
Stroke	3/29 (10.3%)	1/7 (14.3%)	2/22 (9.1%)	1
Pulmonary diseases	6/29 (20.7%)	1/7 (14.3%)	5/22 (22.7%)	1
CCI (median, IQR)	4 (2–6)	5 (2–7)	4 (2.75–6)	0.59
Days of therapy (IQR)	11 (7.5–14.4)	10 (8–14)	11 (7–15)	0.94
Days in hospital (IQR)	48 (29.5–89.5)	49 (42–60)	45 (27–94.5)	0.59
Days on MV	26 (5–37)	34.5 (29.75–54.25)	12 (3–27)	**0.013**
ICU	17/29 (58.6%)	6/7 (85.7%)	11/22 (50%)	0.19
Days from sampling to ATB	5 (2–7.75)	6 (0–14.5)	5 (2–7.75)	0.82
Time to BC clearance	10 (5.25–12.25)	6 (5-)	10 (7–13)	0.46
Source of infection				0.24
HAP/VAP	5/27 (18.5%)	2/6 (33.3%)	3/21 (14.3%)	
BSI	6/27 (22.2%)	2/6 (33.3%)	4/21(19%)	
cIAI	6/27 (22.2%)	0/6	6/21 (28.6%)	
UTI	3/27 (11.1%)	0/6	3/21 (14.3%)	
BSI + HAP/VAP	4/27 (14.8%)	1/6 (16.7%)	3/21 (14.3%)	
SSTI	2/27 (7.4%)	0/6	2/21 (9.5%)	
CSF	1/27 (3.7%)	1/6 (16.7%)	0/21	

**Table 4:** Spearman’s rho correlation between 30-day mortality and other variables.

		95% Confidence Intervals (2-Tailed)		*p*-Value
Thirty-day mortality	Spearman’s rho	Lower	Upper	
Parenteral nutrition	0.27	0.053	0.696	0.023
Mechanical Ventilation	0.315	−0.069	0.618	0.096
Arterial catheter	0.384	0.009	0.664	0.04
Sepsis	0.378	0.002	0.661	0.043
Candidemia	0.354	−0.018	0.64	0.055

## Data Availability

The raw data supporting the conclusions of this article will be made available by the authors on request.
